# Assessing the benefits and harms of direct oral anticoagulants in patients with cancer for the prophylaxis and treatment of venous thromboembolism: a systematic review and meta-analysis

**DOI:** 10.3332/ecancer.2020.1091

**Published:** 2020-08-25

**Authors:** Aakash Desai, Bishal Gyawali

**Affiliations:** 1 Department of Medicine, University of Connecticut, Farmington, CT, USA; 2 Department of Oncology, Queen’s University, Kingston, Canada; 3 Department of Public Health Sciences, Queen’s University, Kingston, Canada; 4 Division of Cancer Care and Epidemiology, Queen’s University, Kingston, Canada

**Keywords:** venous thromboembolism, direct oral anticoagulants, edoxaban, apixaban, rivaroxaban

## Abstract

**Background:**

Direct oral anticoagulants (DOACs) have recently been tested in multiple randomised controlled trials (RCTs) for the prophylaxis and treatment of cancer-associated venous thromboembolism (VTE) leading to changes in guidelines. To quantify the risks and benefits of DOACs in the prophylaxis and treatment of cancer-associated VTE, we performed a systematic review and meta-analysis of published RCTs.

**Methods:**

A systematic search of PubMed, Cochrane Library and Google Scholar databases for all phase-3 RCTs of DOACs in patients with cancer was conducted. Pooled estimates for the cumulative incidence of VTE, recurrent VTE, major bleeding and clinically relevant non-major bleeding (CRNMB) for each arm and pooled hazard ratio (HR) with 95% confidence intervals (CI) for VTE, recurrent VTE, major bleeding, CRNMB and overall survival were calculated by using random-effect model.

**Results:**

Six phase-3 RCTs (*N* = 4341) which studied DOACs in prophylaxis or treatment of cancer-associated VTE were included. DOACs significantly reduced the risk of VTE versus placebo in prophylaxis (5% versus 9%, HR 0.51 and 95% CI:0.32–0.82) and the risk of recurrent VTE versus low-molecular-weight heparin in the treatment setting (4% versus 9%, HR 0.58 and 95% CI: 0.40–0.87) although, at a cost of increased risk of major bleeding (HR 1.46 and 95% CI: 1.0–2.12) or CRNMB (HR 1.42 and 95% CI: 1.10–1.81), there was no effect on survival (HR 1.01 and 95% CI: 0.85–1.20).

**Conclusion:**

In this meta-analysis, we found that DOACs not only significantly decreased the risk of VTE or recurrent VTE in patients with cancer but also significantly increased the risk of bleeding and CRNMB, with neither beneficial nor detrimental effects on survival. The quantification of these benefits and risks will assist in individualised shared decision-making.

## Introduction

Although venous thromboembolism (VTE) is one of the most common complications and causes of death in patients with cancer, the prophylaxis and treatment of VTE in patients with cancer have not been straightforward for a number of reasons. Patients with cancer are not only at a higher risk of VTE but also at a higher risk of bleeding due to thrombocytopenia, organ or vascular invasion [1]. Therefore, balancing the risks and benefits of anticoagulation needs a thorough understanding of the underlying objective evidence in addition to clinical judgement.

A common issue in the anticoagulation of patients with cancer is the need for long-term treatment with low-molecular-weight heparin (LMWH) injections and its associated quality of life burden. Furthermore, the duration of treatment is unknown. In ambulatory patients, the burden of prophylactic anticoagulation has usually been deemed to outweigh the benefits. However, a number of studies have been published recently assessing the role of direct oral anticoagulants (DOACs) in patients with cancer, both in the prophylaxis and treatment settings. This could change the balance of risks–benefits by providing a non-injection option for anticoagulation. Indeed, the American Society of Clinical Oncology (ASCO) has already changed its guidelines, now recommending thromboprophylaxis with apixaban, rivaroxaban, or LMWH to selected high-risk outpatients with cancer and rivaroxaban or edoxaban as options for VTE treatment [2]. The addition of rivaroxaban, apixaban and edoxaban to the treatment armamentarium is a major change from the previous guidelines which recommended LMWH as the treatment of choice or did not deem the benefits to outweigh the therapeutic burden for prophylaxis in ambulatory setting.

To support an informed shared decision-making, it is important to understand and quantify the benefits and risks of DOACs in patients with cancer, both in the prophylactic and therapeutic settings. We, therefore, performed a systematic review and meta-analysis of randomised controlled trials (RCTs) evaluating DOACs in prophylaxis or treatment of cancer-associated VTE to provide a summary estimate of benefits and harms of their use.

## Methods

This systematic review and meta-analysis were conducted in accordance with the Preferred Reporting Items for Systematic Reviews and Meta-Analyses reporting guidelines [3].

### Study selection

We conducted a systematic search of PubMed, Cochrane Library and Google Scholar for all phase-3 RCTs of direct oral anticoagulants using the search terms direct oral anticoagulants or oral anticoagulants or DOACs or NOACs or OACs or rivaroxaban or apixaban or edoxaban and cancer. We excluded non-trial studies, non-RCTs and studies conducted in population that was not specific to cancer and included only phase-3 RCTs which studied the use of DOACs in prophylaxis or treatment of VTE in cancer patients. Both placebo control and active control RCTs were included. The search was conducted on March 31, 2020.

### Data extraction

This study was not submitted for institutional review board approval because it did not involve individual patient information, and all data extractions were made from publicly available published articles. After title and abstract screening by the two authors, the full texts of potentially relevant studies were downloaded, the relevant data were independently extracted from published reports by the two authors and any discrepancy was resolved by verifying with the publication and mutual consensus.

We collected key trial characteristics: study name, year of publication, treatment setting, control arm, median duration of treatment and sample size for efficacy and safety. We used the intention-to-treat population for assessing efficacy and survival outcomes and the safety population for assessing safety outcomes.

The data on efficacy and safety population, number of VTE or recurrent VTE along with the hazard ratio (HR) and corresponding 95% confidence interval (CI) were extracted from included publications. Similar data were extracted for deaths, major bleeding and clinically relevant non-major bleeding (CRNMB). For the SELECT-D trial, the HR and 95% CI for OS were not available in the publication and were obtained by contacting the corresponding author.

### Endpoints

The primary efficacy outcome was the incidence of VTE for prophylaxis trials and the incidence of recurrent VTE for treatment trials. The primary safety outcome was the incidence of major bleeding. We also assessed the effect of treatment on survival (deaths from any cause) as well as the incidence of CRNMB.

### Statistical analysis

The results of systematic review are presented descriptively. For the quantitative meta-analysis, the summary estimates for the cumulative incidence of VTE or recurrent VTE, major bleeding or CRNMB were estimated by pooling the incidence proportions across the trials. As different patients have received treatment for different time periods, the HR provides a better estimate than the risk ratio (RR) by accounting for the time differences. A RR can be biased for a time-to-event outcome because it depends on when the events were measured, whereas an HR provides an estimate of the risk at any given point in time, assuming that the proportional hazard model holds true.[4] In other words, whilst HR provides information on instantaneous risk, a RR provides information on cumulative risk [5]. Thus, we used HR to estimate the risks. The summary hazard ratio and 95% confidence intervals for VTE or recurrent VTE, overall survival (OS), major bleeding and CRNMB were obtained by pooling the HR and 95% CI for each parameter for each RCT. All the pooled estimates were obtained by using random-effect model to account for clinical heterogeneity of included studies. Statistically, the assumption of homogeneity was considered to be invalid for the values of *p* < 0.10 for the Cochrane Q statistic, and the inconsistency was quantified with the I^2^ statistic.

The treatment effects on VTE or recurrent VTE were assessed separately for prophylaxis and treatment settings as the intent and the comparator arms were different. However, for the effect on survival or incidence or risks of bleeding, we pooled data across all RCTs but performed subgroup analyses by treatment setting (prophylaxis versus treatment) or DOAC drug type.

All statistical analyses were performed using Stata version 15 (StataCorp). For meta-analysis, metaprop command was used for pooling proportions, and metan command was used for pooling ratios.

## Results

**Figure 1.**
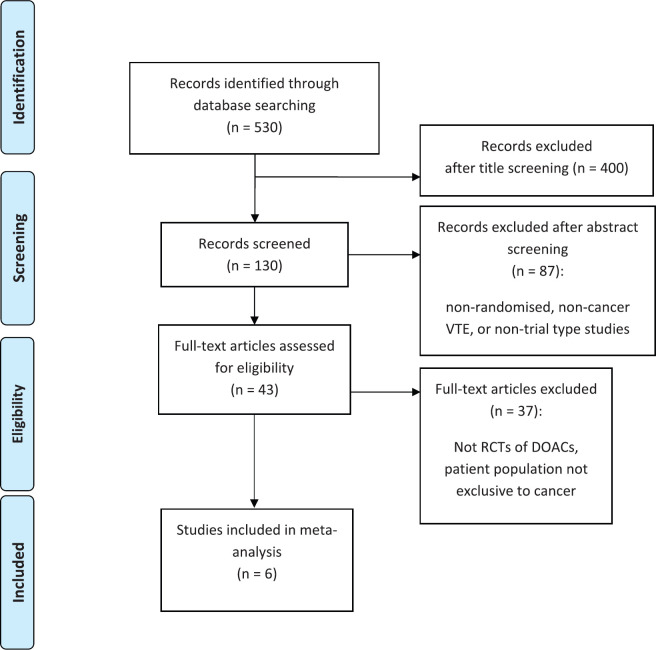
PRISMA diagram.

Of the 530 studies initially identified, 43 were RCTs, of which six (*N* = 4,341) were phase-3 RCTs of DOACs in patients with cancer and were included in the analysis. There were two RCTs (*N* = 1,415) identified in the prophylaxis and four RCTs (*N* = 2,926) in the treatment setting (Figure 1).

## Systematic review

### Anticoagulation for VTE prophylaxis in patients with cancer

Two randomised trials AVERT [6] and CASSINI [7] evaluated the role of DOACs in the prophylaxis of VTE (Table 1). AVERT compared apixaban 2.5 mg BID versus placebo, whereas CASSINI studied rivaroxaban 10 mg OD versus placebo. As shown in Table 1, AVERT showed a statistically significant effect of apixaban on the primary efficacy endpoint of preventing major VTE whilst increasing the risk of major bleeding. CASSINI trial, on the other hand, did not show a significant effect of rivaroxaban on preventing VTE with no significant increase in the risk of major bleeding.

Although both RCTs studied VTE for up to 180 days and enrolled 60%–70% of patients with low VTE risk (Khorana Score 2), there were certain key differences. CASSINI randomised 841 patients, whereas AVERT had a smaller sample size of 563. CASSINI had a higher percentage (54%) of patients with metastatic disease compared to AVERT (25%). In addition, CASSINI had a higher percentage of patients with pancreatic and gastric cancers, and cancers considered to be at a higher risk of VTE. Patients enrolled in CASSINI were also screened prior to randomisation; about 4.5% of those screened were found to have thrombosis and were excluded from the study. Such screening was not performed for enrollment to AVERT trial.

### Anticoagulation for VTE treatment in cancer patients

We found four RCTs which assessed the risks and benefits of DOACs in treating patients with cancer-associated VTE, all versus LMWH:ADAM-VTE [8], CARAVAGGIO [9], HOKUSAI-VTE [10] and SELECT-D [11] enrolling 300, 1,170, 1,050 and 406 patients, respectively (Table 2).

Two RCTs have compared the efficacy and safety of apixaban with dalteparin. The ADAM-VTE trial demonstrated that apixaban did not lead to an increase in major bleeding or CRNMB whilst improving QoL and decreased the rate of recurrent VTE. More recently, CARAVAGGIO demonstrated that apixaban was non-inferior to dalteparin for recurrent VTE, and non-inferiority limit was defined at 2.0 for the upper limit of 95% CI for HR of recurrent VTE. The safety outcomes were not significantly different between the two groups.

HOKUSAI-VTE was also a non-inferiority trial, which showed that edoxaban was non-inferior to dalteparin with regards to recurrent VTE but had a higher risk of major bleeding. The non-inferiority limit for this trial was set at 1.5 for the upper limit of the 95% CI for HR.

The efficacy of rivaroxaban was established by the SELECT-D trial, where rivaroxaban showed a decreased incidence of recurrent VTE over 6 months without significant difference in major bleeding versus dalteparin.

### Meta-analysis (Table 3)

### Effect on VTE

The pooled cumulative incidence for VTE in the prophylactic settings with DOACs was 5% (95% CI: 3%–7%) compared with 9% (95% CI: 7%–11%) with placebo. In the treatment settings, the pooled cumulative incidence of recurrent VTE was 4% (95% CI: 1%–8%) with DOACs compared with 9% (95% CI: 7%–11%) with dalteparin.

**Figure 2.**
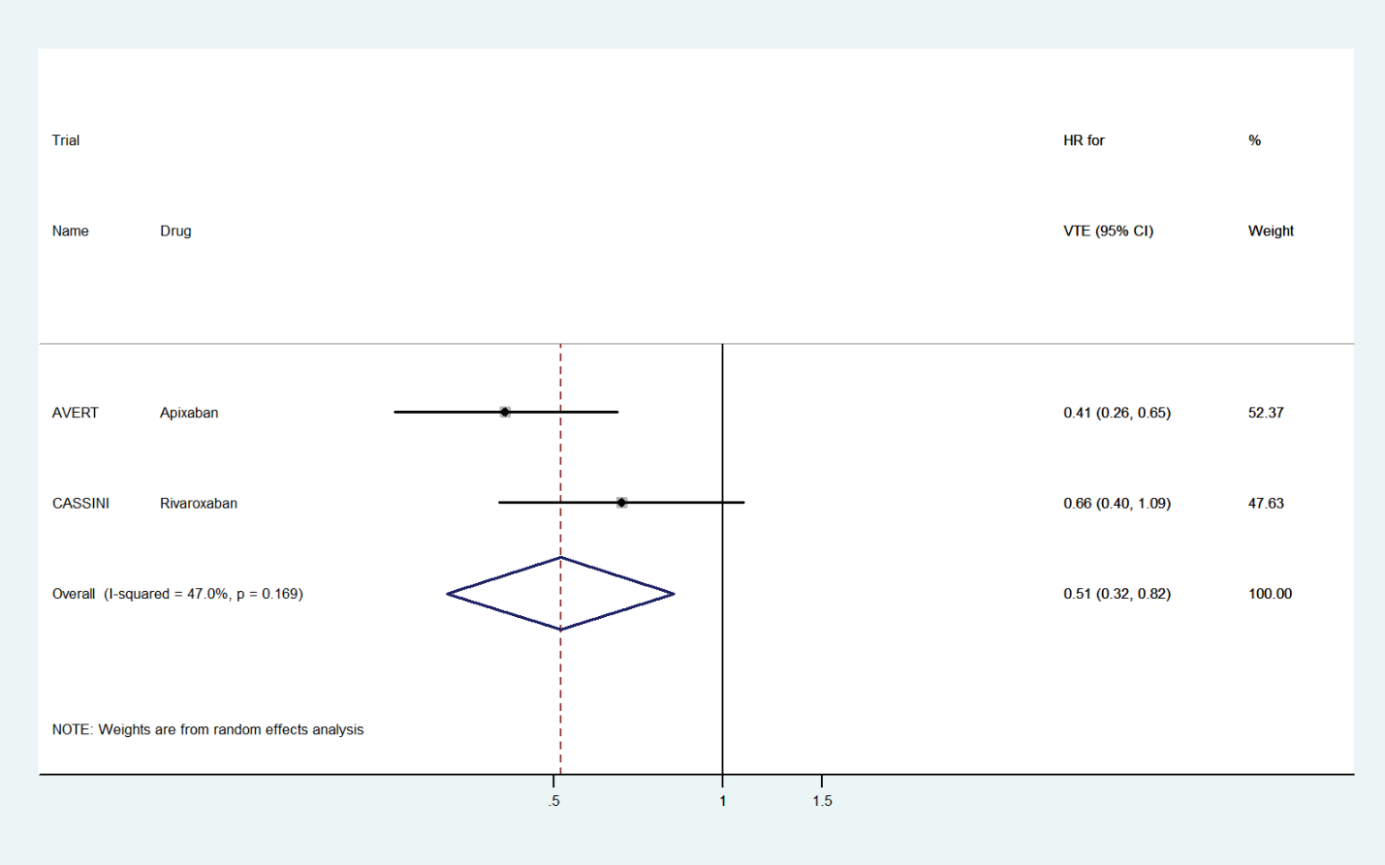
Forest plots of pooled HR for VTE prophylaxis with DOACs versus placebo in patients with cancer.

**Figure 3.**
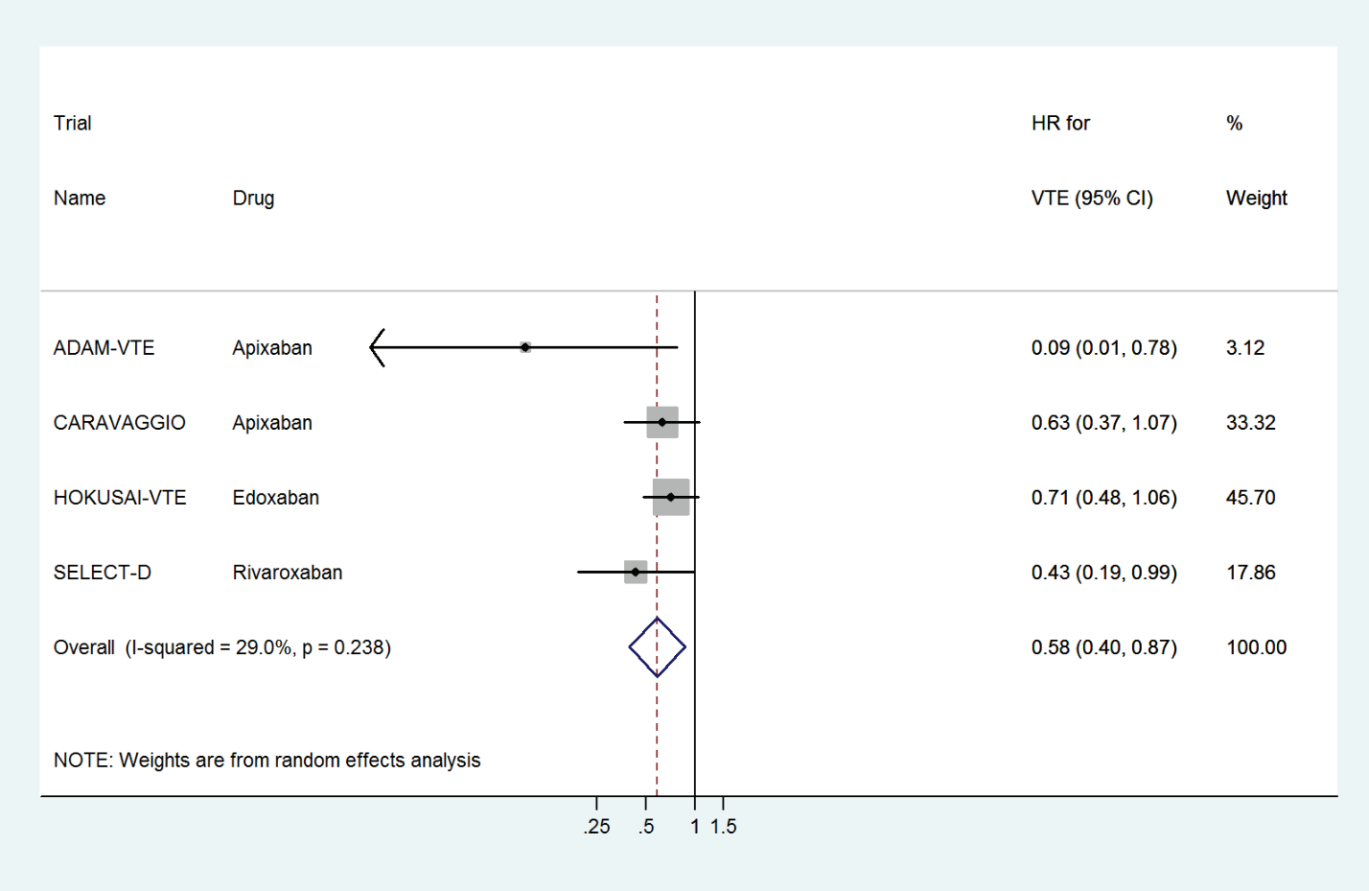
Forest plots of pooled HR for VTE treatment with DOACs versus LMWH in patients with cancer.

The pooled HR for VTE prophylaxis versus placebo was 0.51 (95% CI: 0.32–0.82) (Figure 2) and for preventing recurrent VTE in the treatment setting versus dalteparin was 0.58 (95% CI: 0.40–0.87) (Figure 3).

### Effect on major bleeding

**Figure 4.**
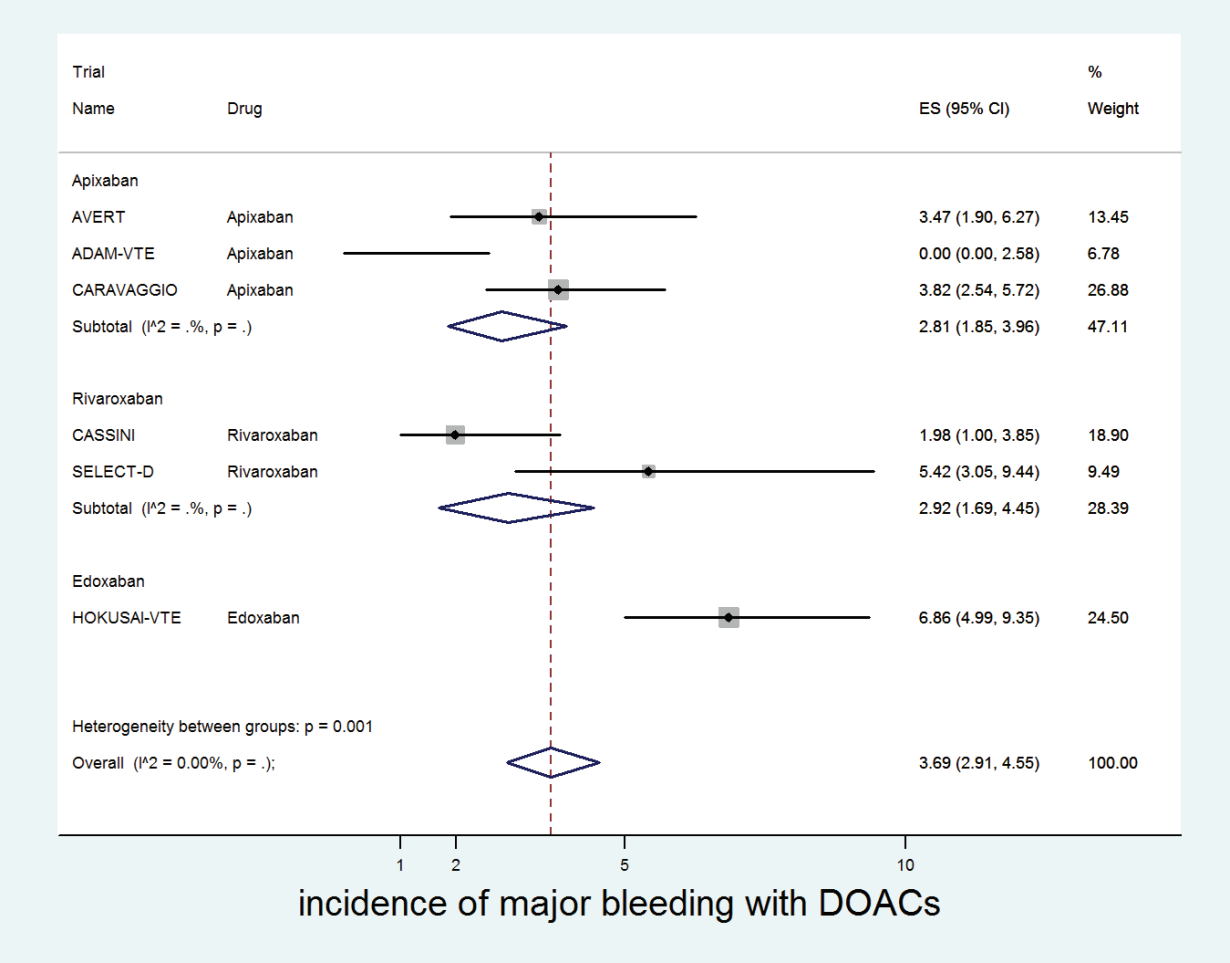
Forest plots of cumulative incidence of Major Bleeding(MB) with DOACs in patients with cancer with subgroup analysis by drug type.

The cumulative incidence of major bleeding with DOACs was 3.7% (95% CI: 2.9%–4.5%) with incidence significantly higher in patients receiving edoxaban (6.8%) versus apixaban (2.8%) or rivaroxaban (2.9%) (*p* for heterogeneity between groups = 0.001, Figure 4).

**Figure 5.**
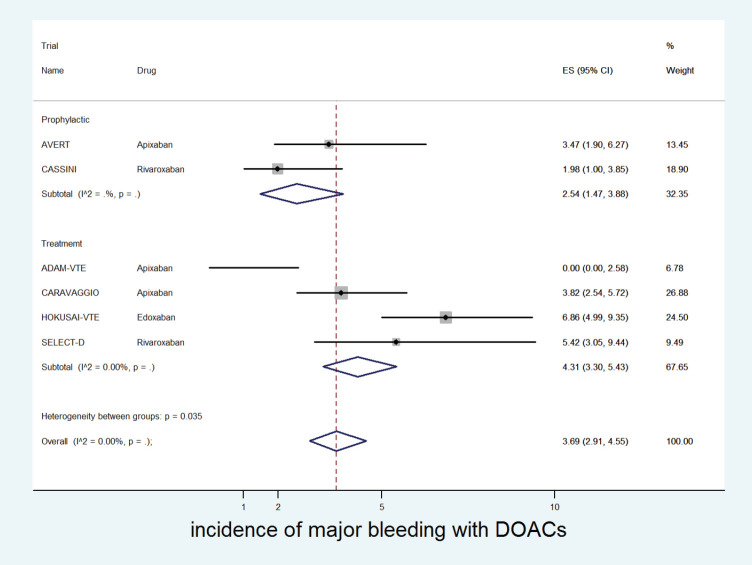
Forest plots of cumulative incidence of Major Bleeding(MB) with DOACs in patients with cancer with subgroup analysis by treatment setting.

The incidence of major bleeding with DOACs was significantly higher in the treatment setting (4.3%, 95% CI: 3.3%–5.4%) versus prophylactic setting (2.5%, 95% CI: 1.5%–3.9%) (*p* for heterogeneity between groups = 0.035, Figure 5). The incidence of major bleeding with placebo was 1% (95% CI: 0%–2%) and that with dalteparin was 3% (95% CI: 2%–4%).

**Figure 6.**
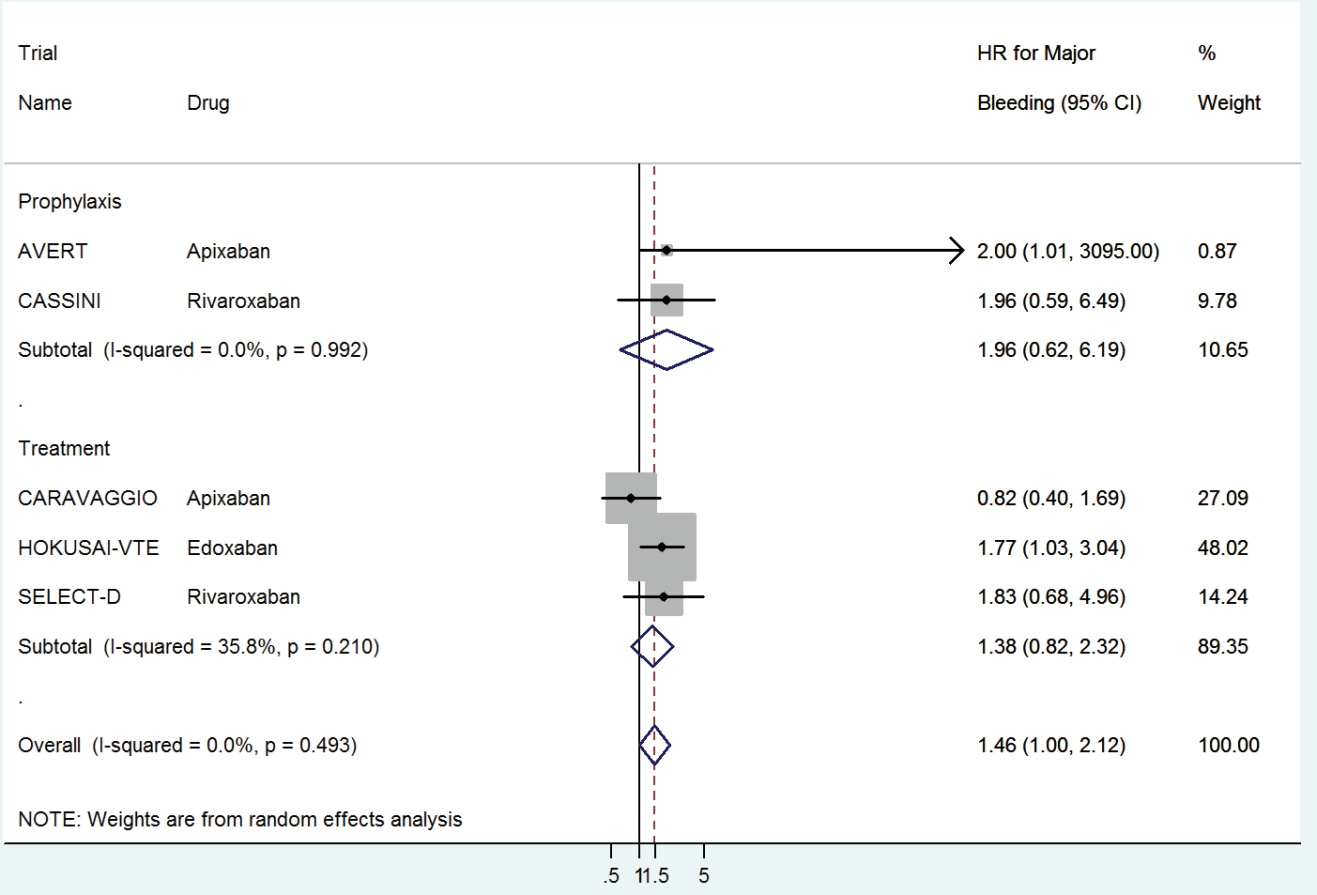
Forest plots of pooled HR for Major Bleeding(MB) with DOACs versus control in patients with cancer with subgroup analysis by treatment setting.

**Figure 7.**
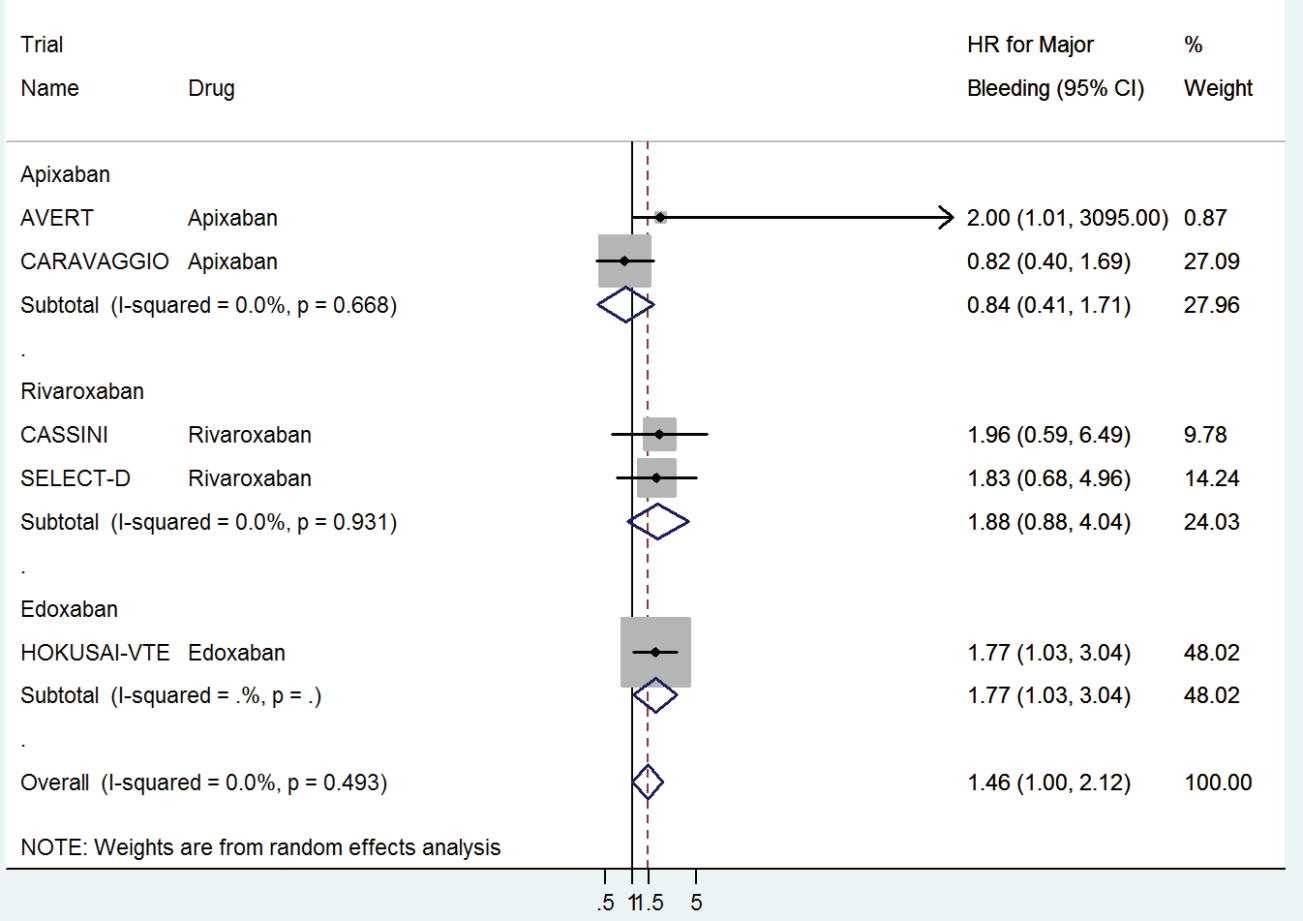
Forest plots of pooled HR for Major Bleeding(MB) with DOACs versus control in patients with cancer with subgroup analysis by drug type.

The HR for major bleeding with DOACs versus control was significantly higher with a pooled HR of 1.46 (95% CI: 1.0–2.12) with no significant heterogeneity in prophylactic (HR 1.96, 95% CI: 0.62–6.19) versus treatment trials (HR 1.38, 95% CI: 0.82–2.32) (Figure 6) or with drug type (Figure 7).

### Effect on CRNMB

**Figure 8.**
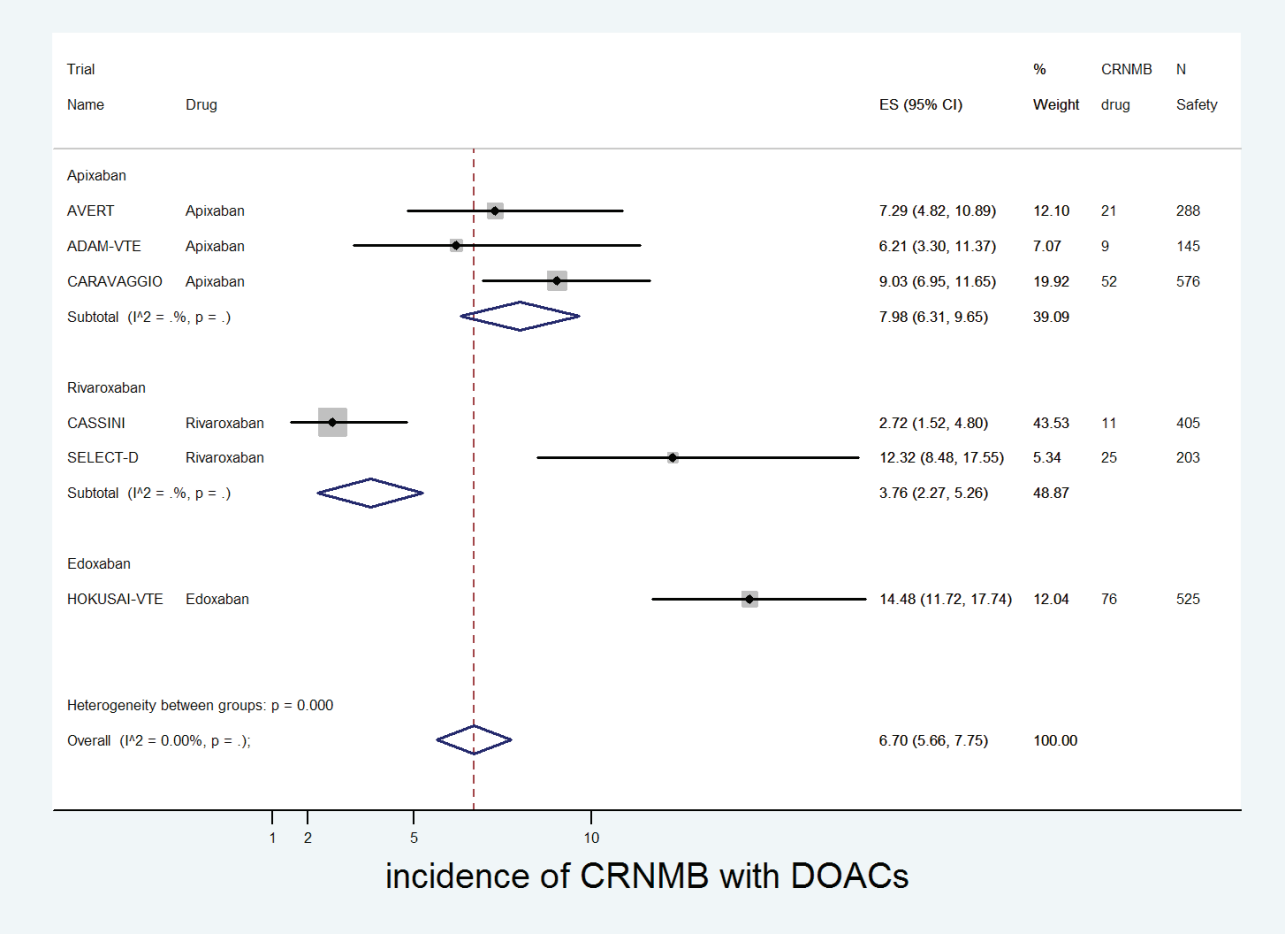
Forest plots of cumulative incidence of Clinically Relevant Non-Major Bleeding(CRNMB) with DOACs in patients with cancer with subgroup analysis by drug type.

**Figure 9.**
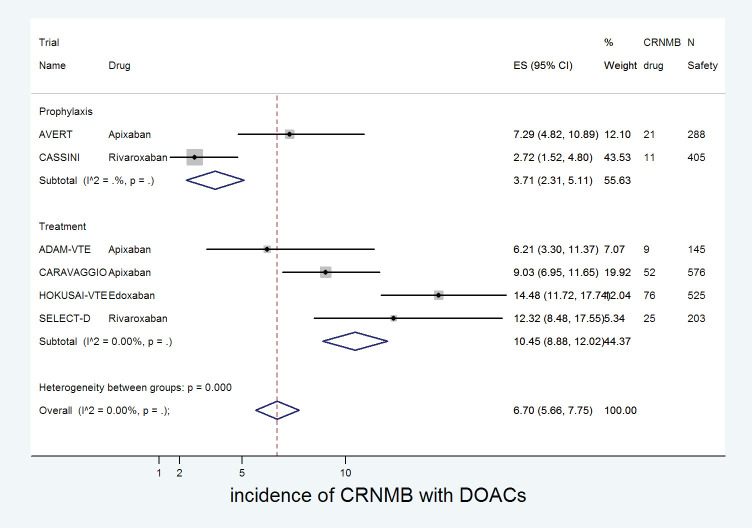
Forest plots of cumulative incidence of Clinically Relevant Non-Major Bleeding(CRNMB) with DOACs in patients with cancer with subgroup analysis by treatment setting.

The cumulative incidence of CRNMB with DOACs was 6.7% (95% CI: 5.7%–7.8%) with incidence significantly higher in edoxaban (14%) versus apixaban (8%) or rivaroxaban (4%) (*p* for heterogeneity between groups < 0.001, Figure 8). The incidence of CRNMB with DOACs was also higher in the treatment setting (10.5%) versus prophylactic setting (3.7%) (Figure 9). The incidence of CRNMB with placebo was 3% (95% CI: 1%–4%) and that with dalteparin was 6% (95% CI: 3%–10%).

**Figure 10.**
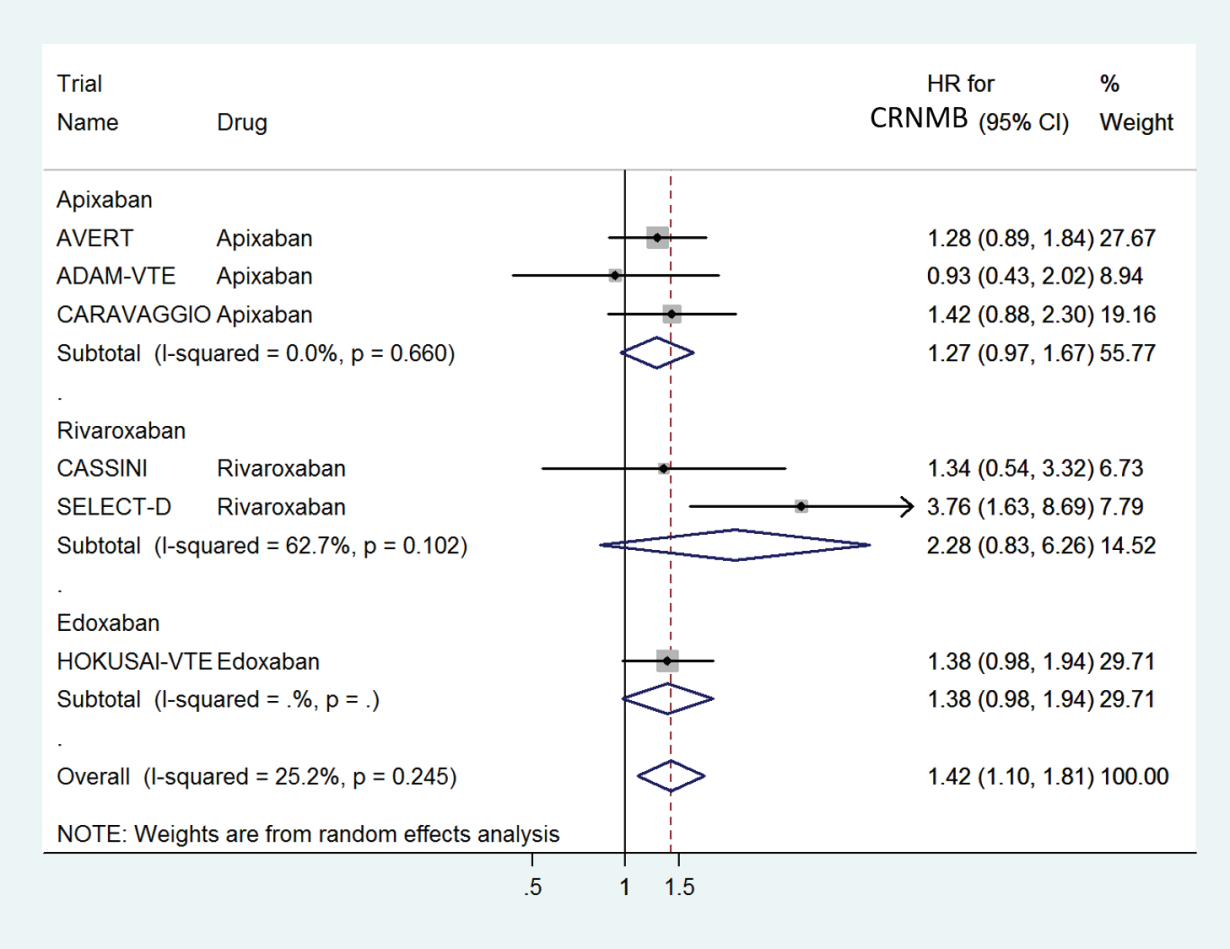
Forest plots of pooled HR for Clinically Relevant Non-Major Bleeding(CRNMB) with DOACs versus control in patients with cancer with subgroup analysis by drug type.

**Figure 11.**
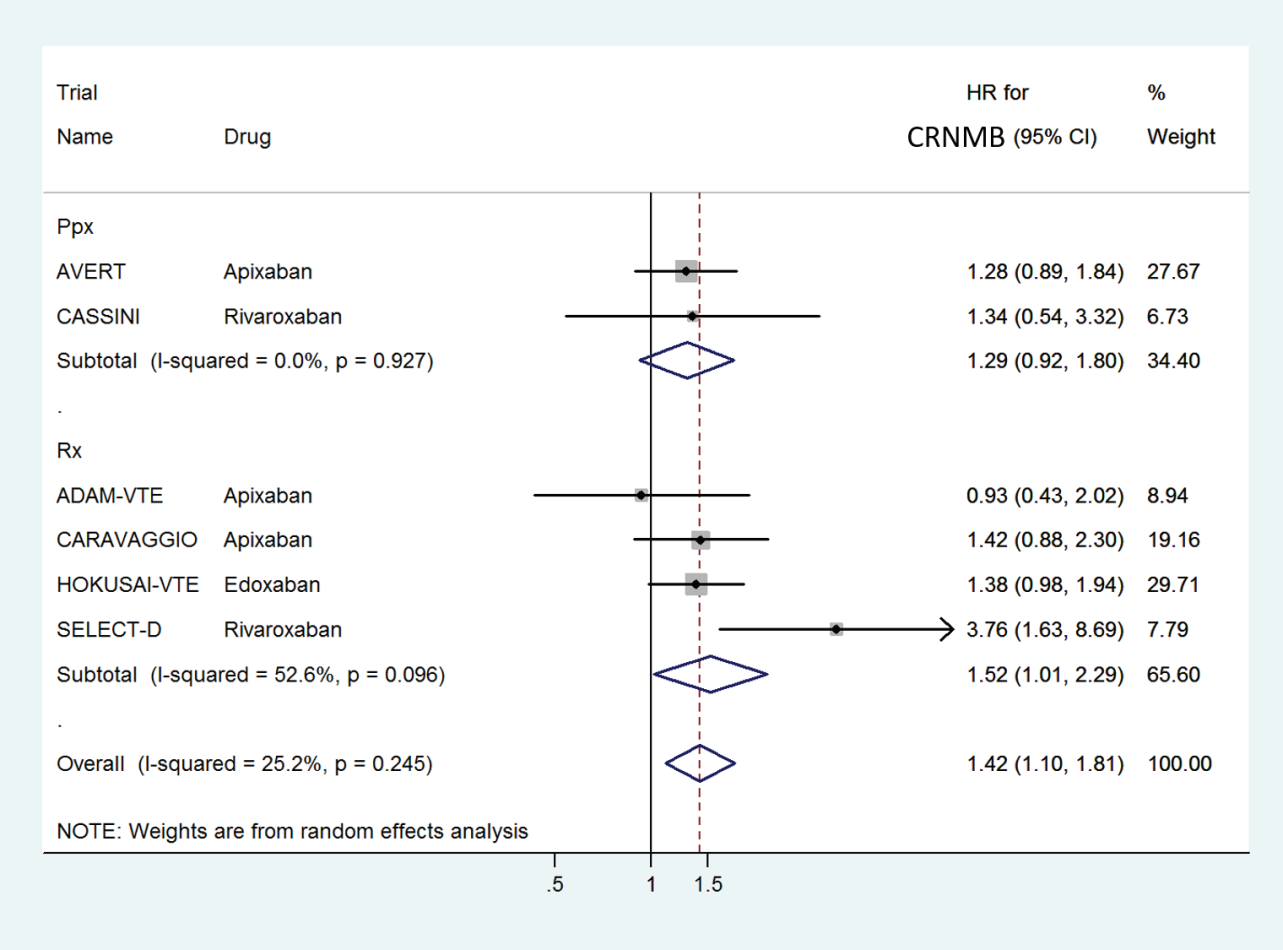
Forest plots of pooled HR for Clinically Relevant Non-Major Bleeding(CRNMB) with DOACs versus control in patients with cancer with subgroup analysis by treatment setting.

The HR for CRNMB with DOACs versus control was significantly higher with a pooled HR of 1.42 (95% CI: 1.10–1.81) with non-significant heterogeneity with drug type (Rivaroxaban 2.28, Edoxaban 1.38 and Apixaban 1.27) (Figure 10) or in prophylactic (HR 1.29) versus treatment trials (HR 1.52) (Figure 11).

### Effect on OS

**Figure 12.**
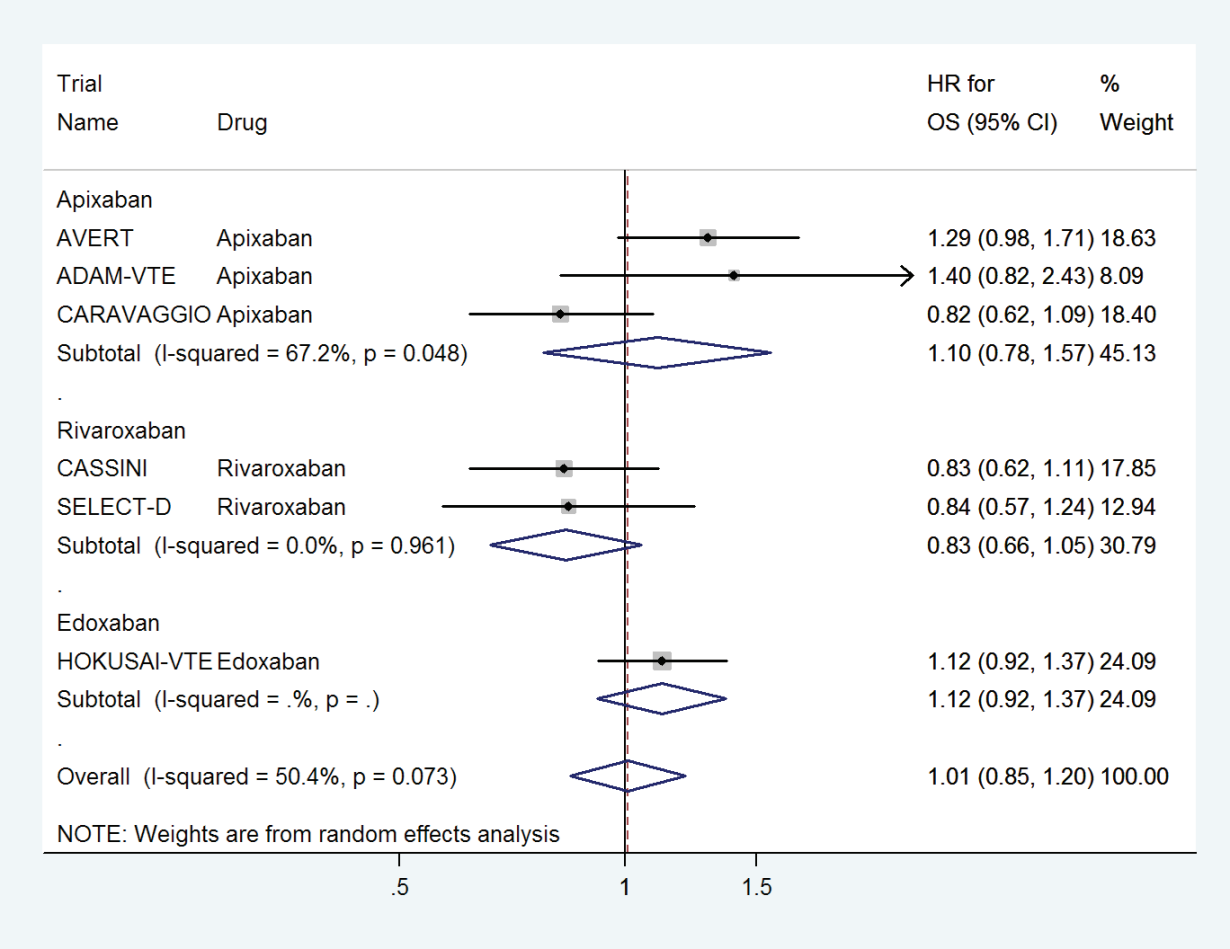
Forest plots of pooled HR for Overall Survival(OS) with DOACs versus control in patients with cancer with subgroup analysis by drug type.

**Figure 13.**
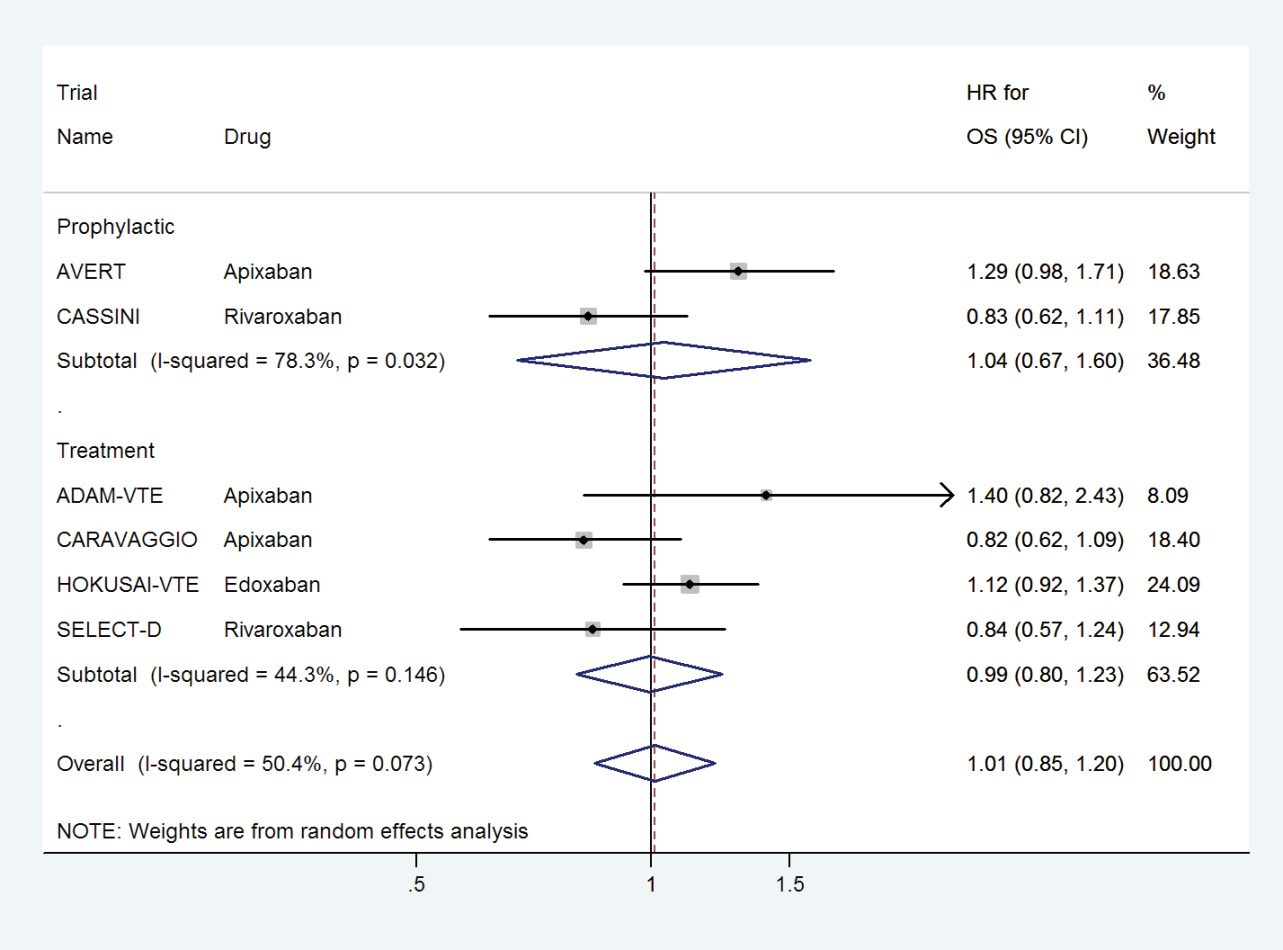
Forest plots of pooled HR for Overall Survival(OS) with DOACs versus control in patients with cancer with subgroup analysis by treatment setting.

DOACs did not have any effect on overall survival with a pooled HR of 1.01 (95% CI: 0.85–1.20) with some heterogeneity based on drug type (Figure 12) or treatment settings (Figure 13).

## Discussion

In this systematic review and meta-analysis, we found that the use of DOACs in patients with cancer had important trade-offs both in the prophylactic and therapeutic settings. Whilst the use of DOACs did decrease the risk of developing VTE or recurrent VTE, the use of DOACs did not confer any survival advantage but came at a cost of increase in the risk of major bleeding as well as CRNMB which did not translate to increased mortality either. The pooled data presented in this study will support the patient understanding of the risks and benefits and support as a tool for shared decision-making.

Although it was recognised that some patients are at a higher risk of developing VTE, prophylactic anticoagulation was not recommended for ambulatory patients until recently as the burden of LMWH injections was considered higher than the benefits of anticoagulation. This recommendation is now being reconsidered after the publication of AVERT and CASSINI trials. Although the meta-analysis does support the decrease in the risk of developing VTE with DOACs by as much as 50%, it also shows that there is nearly as much increase in the risks of major bleeding and CRNMB. It is also important to note that CASSINI alone did not lead to a statistically significant decrease in the risk of VTE. Since CASSINI screened the participants for occult thrombosis before enrolment but AVERT did not, this also calls into question the positive effects seen in AVERT because if a high percentage of the patients did have latent VTE, the effect seen in AVERT would simply be a therapeutic effect of treating occult VTE rather than the prophylactic effect of preventing VTE.

For the treatment of confirmed VTE in patients with cancer, LMWH was the anticoagulant of choice until recently based on superior outcomes versus vitamin K antagonists [12]. The DOACs represent an attractive option in this setting as well because they are taken orally at fixed doses without the need for laboratory monitoring. The pooled estimates suggest that the DOACs not only provide convenience of being an oral agent but also have better VTE relapse prevention effects than LMWH; however, this effect was also associated with increased risks of bleeding. Furthermore, although the non-inferiority design is justified given the convenience of oral therapy versus LMWH injection for 6 months, the non-inferiority margins set at 2.0 or 1.5 in the trials can be considered too lenient.

The lack of OS benefit with DOACs for cancer-associated VTE, both in individual RCTs and the pooled analysis, is perplexing because VTE is considered to be one of the most common causes of death in patients with cancer. If indeed the decrease in VTE-related deaths was counterbalanced by the increase in bleeding-related deaths in the long term, it would either imply the need for a further refinement of therapy duration or that all VTEs may not be lethal. If the latter were true, one could argue that preventing VTE in itself may not be a clinical endpoint, and future trials in the prophylactic setting may need to consider other endpoints such as prevention of pulmonary embolism or death due to VTE.

Although we considered only major bleeding and CRNMB for the assessment of harms, another important toxicity to consider in patients with cancer is financial toxicity [13]. This is all the more important to consider in the absence of demonstrable survival benefit, especially for the treatment of VTE in patients with incurable metastatic cancer with a limited lifespan or for ambulatory patients being considered for prophylaxis of VTE. The meta-analysis provides data that can be used to estimate the cost-effectiveness of the DOACs versus placebo for prophylaxis or LMWH for the treatment of cancer-associated VTE. For patients who are already struggling with financial toxicity due to the costs of cancer treatment, it is important to consider the costs of drugs as well as the costs of managing their side effects such as major bleeding for informed decision-making.

Real-world studies have revealed that DOACs are prescribed for up to one-fifth of patients with cancer-associated VTE [14]. The pooled analysis argues for an individualised approach to VTE prophylaxis and treatment in patients with cancer. The decision to anticoagulate and the choice of anticoagulant should take into account the risks of thrombosis, the risks of bleeding, stage of the disease, expected life expectancy, expected duration of anticoagulation and the costs and quality of life. Since both the decrease in the risk of thrombosis and increase in the risks of bleeding are significant with DOACs, whilst OS remains unchanged, there is probably no right ‘fit-for-all’ answer.

Currently, the most recent 2020 ASCO guidelines [2] recommend thromboprophylaxis with apixaban or rivaroxaban, for selected high-risk outpatients with cancer (Khorana score of 2 or higher prior to starting a new systemic chemotherapy regimen). ASCO also recommends using rivaroxaban or edoxaban for VTE treatment. This guideline was published prior to the publication of CARVAGGIO trial. The 2019 NCCN guidelines [15] describe using LMWH for VTE treatment as category 1 recommendation, whereas DOAC use is a category 2A recommendation. For prophylactic anticoagulation in inpatient or outpatient setting, NCCN provides recommendations for LMWH, aspirin or warfarin but does not provide guidance on DOAC use. The European Society for Medical Oncology has not updated the guidelines for cancer-associated VTE since 2011 [16]. This meta-analysis that includes information from the most recent CARVAGGIO trial provides objective pooled data for benefits and harms that can help update future versions of the guidelines.

There are some caveats to consider in the interpretation of our study. We included only RCTs in our analysis (and excluded retrospective or cohort studies) because RCTs provide the highest level of evidence and would also be less heterogeneous, allowing us to pool data to provide summary estimates for incidences and risks. In addition, although dabigatran is a DOAC, there are no RCTs of dabigatran specifically conducted for the population of patients with cancer. Therefore, the results are not generalizable to dabigatran. The results of subgroup analysis in this study should be considered only hypothesis generating, especially in those results where there was no statistical evidence of heterogeneity. Furthermore, the heterogeneity in the dose and duration of treatment should be considered when interpreting the subgroup findings. Another important information for clinical decision making would be to assess the risks of bleeding categorised by tumour types since certain cancers such as those of gastrointestinal origin are known to be more at risk for bleeding. Indeed, the enrollment of patients with GI cancers to the SELECT-D trial was stopped by the data safety monitoring committee due to the concerns of increased bleeding. However, none of the RCTs provided enough information on bleeding risks by tumour types to allow us to do a subgroup analysis, and only two RCTs provided some information in this regard: amongst patients with gastrointestinal cancers, the risk of MB with DOACs was significantly increased with edoxaban versus dalteparin in HOKUSAI ( 13.2% versus 2.4%, *p* value for interaction based on the presence or absence of gastrointestinal cancers = 0.0169) and with rivaroxaban versus dalteparin in SELECT-D (36% versus 11%). Finally, longer treatment duration with DOACs versus dalteparin could result in the higher rates of bleeding and lower rates of VTE, but the median durations of treatment for both arms were similar in all included RCTs except the HOKUSAI-VTE trial. For the same reason, we decided not to pool a relative risk or risk ratio estimates because the treatment durations were different for different patients and, therefore, pooled hazard ratio would provide a better metric as has been explained earlier in the 'Methods' section.

## Conclusion

In conclusion, the decision regarding the use of DOACs for prophylaxis or treatment of cancer-associated VTE should be made on an individualised basis given significant beneficial effects of DOACs on the prevention of primary or recurrent VTE as well as significant detrimental effects on increased risks of major bleeding and CRNMB, with no significant beneficial or detrimental effects on survival. This meta-analysis provides pooled estimates on the incidence and risks of both VTE and bleeding, to support shared decision-making in clinical practice.

## Role of funding source

There was no funding source for this study. Both the authors had full access to all the data in the study and had the final responsibility for the decision to submit for publication.

## Conflicts of interest

None.

## Figures and Tables

**Table 1.** Anticoagulation for VTE prophylaxis in cancer patients.

Trial	AVERT trial (positive)		CASSINI trial (Negative)	
	Apixaban 2.5 mg BID	Placebo	Rivaroxaban 10 mg OD	Placebo
Country	Canada		Multinational	
Duration	180 days		180 days	
Primary endpoint	Efficacy: Major VTE Safety: Major bleeding		Efficacy: Composite end point Safety: Major Bleeding	
ITT	291	283	420	421
Modified ITT	288	275	-	-
Khorana score 2	186 (64%)	190 (67%)	281(67%)	295(70%)
Median DOT	157 days	155 days	Mean intervention period: 4.3 months	
VTE	12/288 (4.2%)	28/275 (10.2%)	25/420 (6%)	37/421 (8.8%)
HR (CI)	0.41 (0.26–0.65, p < 0.001)		0.66 (0.40–1.09, p = 0.10)	
Deaths	35 (12.2%)	27 (9.8%)	84	100
HR (CI)	1.29 (0.98–1.71)		0.83 (0.62–1.11)	
Safety N	288	275	405	404
Major bleeding	10/288 (3.5%)	5/275 (1.8%)	8/405 (2%)	4/404 (1%)
HR (CI)	2.00 (1.01–3.95, p = 0.046)		1.96 (0.59–6.49)	
Clinically relevant non-major bleeding	21 (7.3%)	15 (5.5%)	11	8
HR (CI)	1.28 (0.89–1.84)		1.34 (0.54–3.32)	
Screened at the beginning	No		Yes, 4.5% had thrombosis	
% Metastatic disease	24.8%		54.5%	
Most common tumour	Lymphoma (25%) and gynaecological (26%)		Pancreatic (33%) and gastric (21%)	

**Table 2.** Treatment RCTs.

	ADAM-VTE Trial		CARAVAGGIO Trial		HOKUSAI-VTE		SELECT-D	
	Apixaban	Dalteparin	Apixaban	Dalteparin	Edoxaban	Dalteparin	Rivaroxaban	Dalteparin
Dose	10 mg BID × 7 days, then 5 mg BID × 6 months	200 IU/kg × 1 month, then 150 U/kg daily	10 mg BID × 7 days, then 5 mg BID × 6 months	200 IU/kg × 1 month, then 150 U/kg daily	LMWH x 5 days, then 60 mg OD	200 IU/kg × 1 month, then 150U/kg	15 mg BID × 3 weeks, then 20 mg OD × 6 months	200 IU/kg × 1 month, then 150 U/kg
Primary Endpoint	Primary safety: Major bleeding secondary efficacy: Thromboembolic events		Efficacy: Recurrent VTE Safety: Major bleeding		Composite of recurrent VTE/major bleeding		VTE recurrence over 6 months	
Non-inferiority limit	N/A		2.00 UL of 95% CI		1.5 UL of 95% CI for primary outcome		N/A	
ITT	150	150	585	585	525	525	203	203
Modified ITT	145	142	576	579				
Median DOT	5.78 months	5.65 months	178 days	175 days	211 days	184 days	5.9	5.8
Recurrent VTE	1/145 (0.7%)	9/142 (6.3%)	32/576 (5.6%)	46/579 (7.9%)	41/525 (7.9%)	59/525 (11.3%)	8/203 (4%)	18/203 (11%)
HR (CI)	0.099 (0.013–0.780, *p* = 0.03)		0.63 (0.37–1.07, *p* < 0.001)		0.71 (0.48–1.06, *p* = 0.006)		0.43 (0.19–0.99)	
Deaths	23/145 (16%)	15/142 (11%)	135/576 (23.4%)	153/579 (26.4%)	206/525 (39.5%)	192/525 (36.6%)	48/203 (23.6%)	56/203(27.6%)
HR (CI)	1.40 (0.82–2.43, *p* = 0.30)		0.82 (0.62–1.09)		1.12 (0.92–1.37)		0.84 (0.57–1.24)	
Major bleeding	0/145 (0%)	2/142 (1.4%)	22/576 (3.8%)	23/579 (4%)	36/525 (6.9%)	21/525 (4.0%)	11/203 (4%)	6/203 (6%)
HR (CI)	Not estimable		0.82 (0.40–1.69, *p* = 0.6)		1.77 (1.03–3.04)		1.83 (0.68–4.96)	
Clinically relevant non-major bleeding	9/145 (6.2%)	7/142 (4.9%)	52/576 (9%)	35/579(6.0%)	76/525 (14.6%)	58/525 (11.1%)	25/203(12.3%)	7/203(3.4%)
HR (CI)	0.931 (0.43–2.02, *p* = 0.88)		1.42 (0.88–2.30)		1.38 (0.98–1.94)		3.76 (1.63–8.69)	
% Metastatic disease	66%		68%		53%		58%	
Most common tumour	Lung (21.8%), Colorectal (19.6%)		N/A		N/A		Colorectal (25%), Lung (11%–12%)	

**Table 3.** Pooled analysis.

	Pooled incidence with DOACs versus control	Pooled HR	Heterogeneity statistics for HR
Prophylactic VTE	5% (3%–7%) versus 9% (7%–11%)	0.51 (0.32–0.82)	I2 = 47%, *p* = 0.169
Recurrent VTE	4% (1%–8%) versus 9% (7%–11%)	0.58 (0.40–0.87)	I2 = 29%, *p* = 0.238
Major bleeding	4% (3%–5%) versus 2% (1%–4%)	1.46 (1.00–2.12)	I2 = 0%, *p* = 0.493
CRNMB	7% (5%–8%) versus 5% (3%–8%)	1.42 (1.10–1.81)	I2 = 25%, *p* = 0.245
Overall Survival	NA	1.01 (0.85–1.20)	I2 = 50%, *p* = 0.073
